# Supplementary material for: Discovery of first-in-class nanomolar inhibitors of heptosyltransferase I reveals a new aminoglycoside target and potential alternative mechanism of action
Source: Sci Rep. 2022 May 4;12:7302. doi: 10.1038/s41598-022-10776-x (PMC9068772; doi:10.1038/s41598-022-10776-x)
Supplement: Supplementary file 1 — Supplementary Information. [file 41598_2022_10776_MOESM1_ESM.pdf]

Supplemental Information

Inhibiting HepI

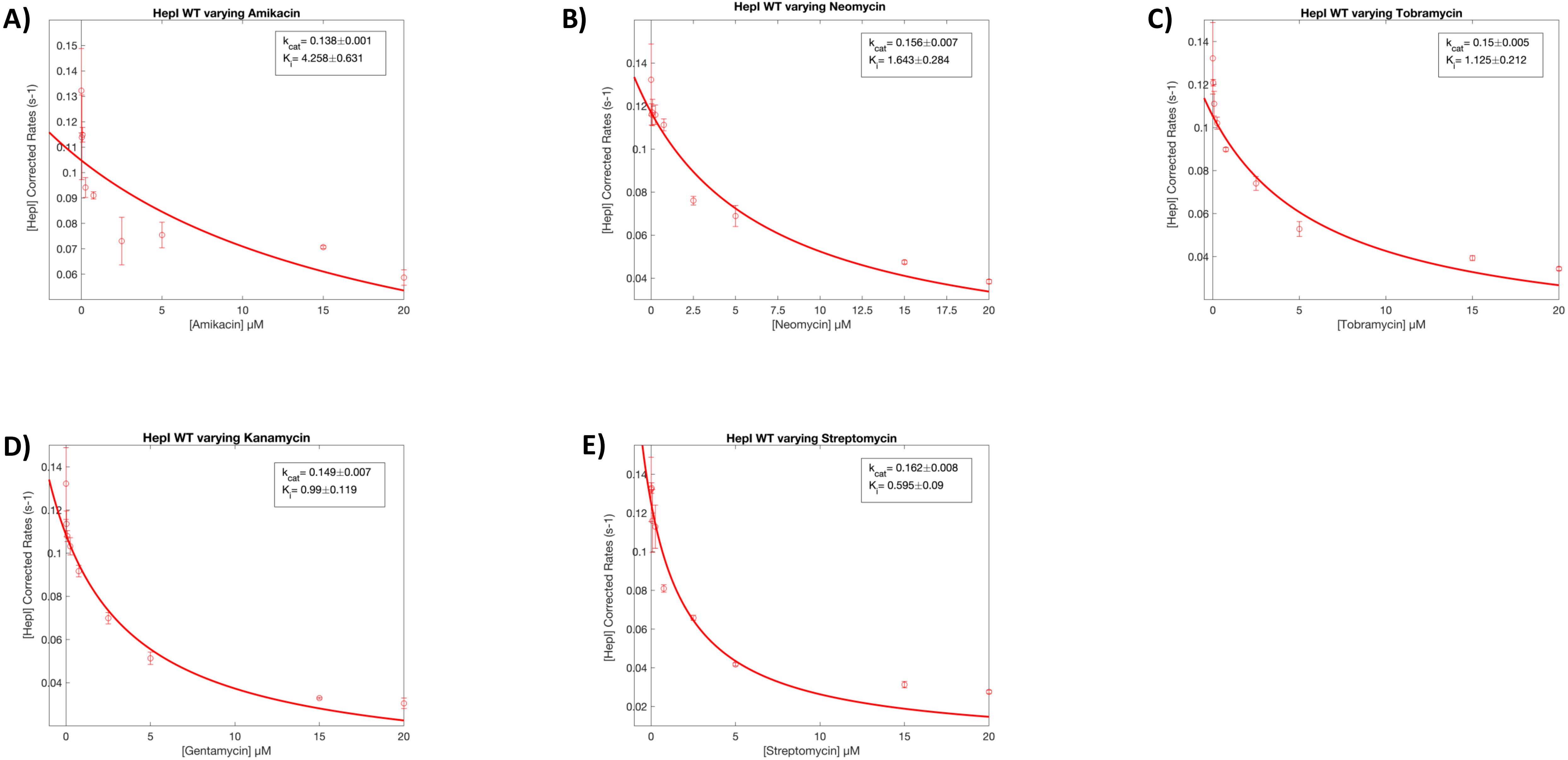

Supplemental Figure 1: Inhibition kinetic curves for HepI in the presence of **A)** Amikacin, **B)** Neomycin, **C)** Tobramycin, **D)** Kanamycin B, or **E)** Streptomycin.

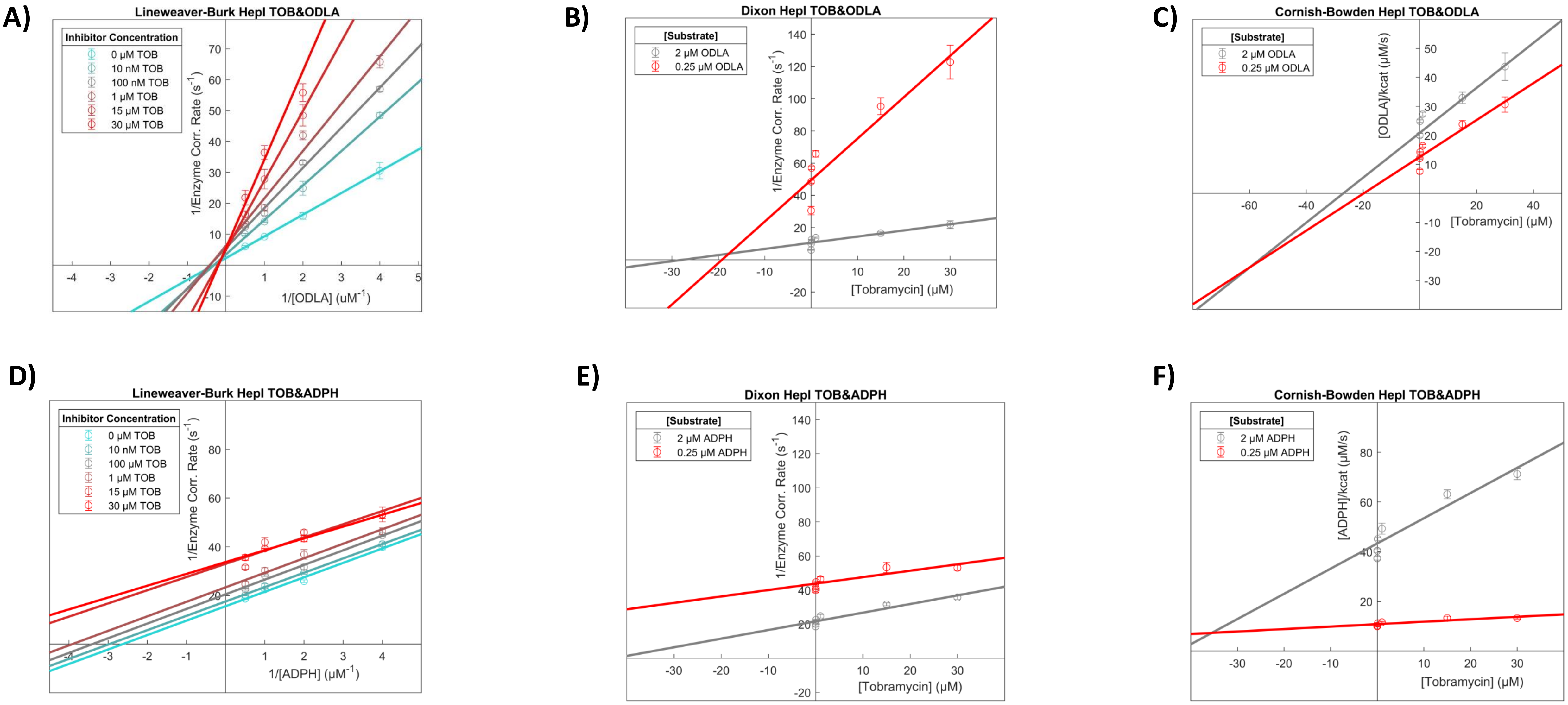

Supplemental Figure 2: Double reciprocal plots of HepI kinetics in the presence of tobramycin with varying **A-C)** ODLA or **D-F)** ADPH.

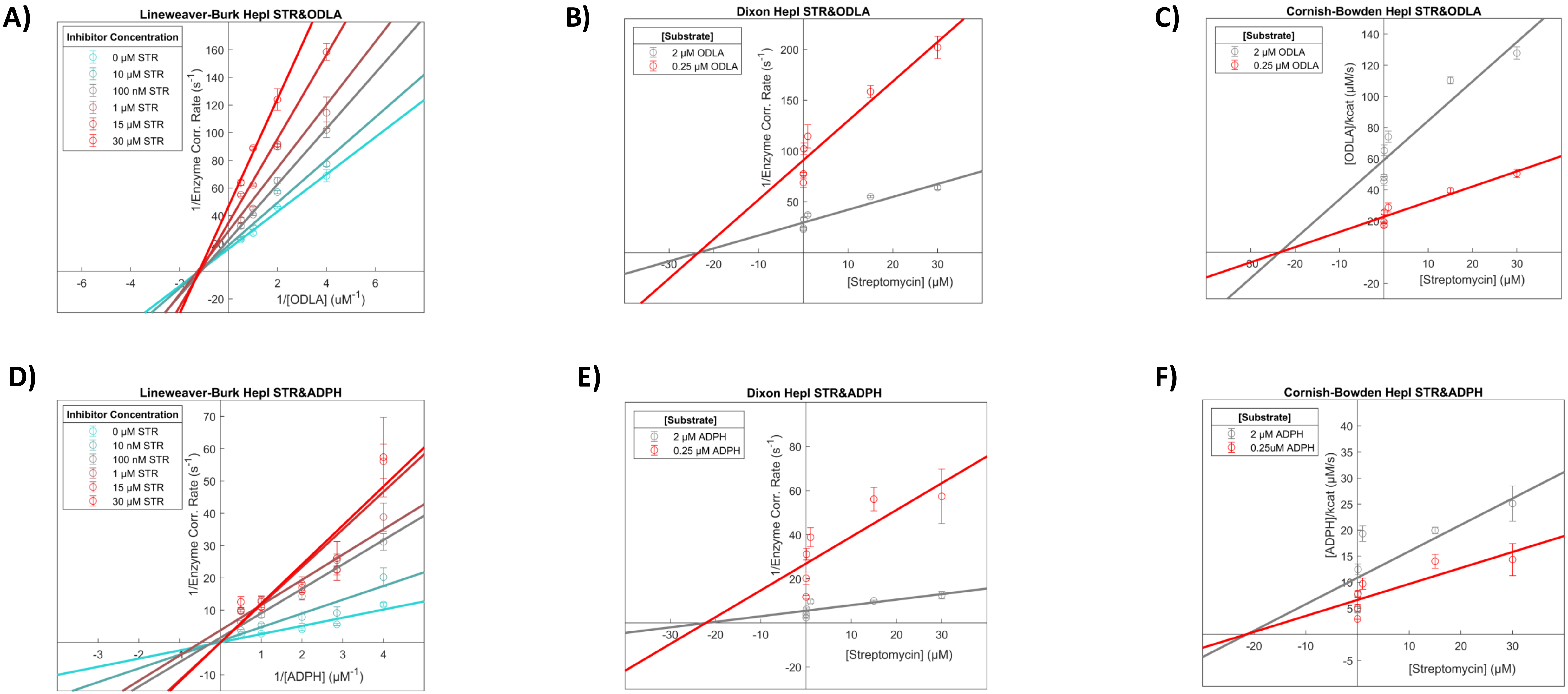

Supplemental Figure 3: Double reciprocal plots of HepI kinetics in the presence of streptomycin with varying **A-C)** ODLA or **D-F)** ADPH.

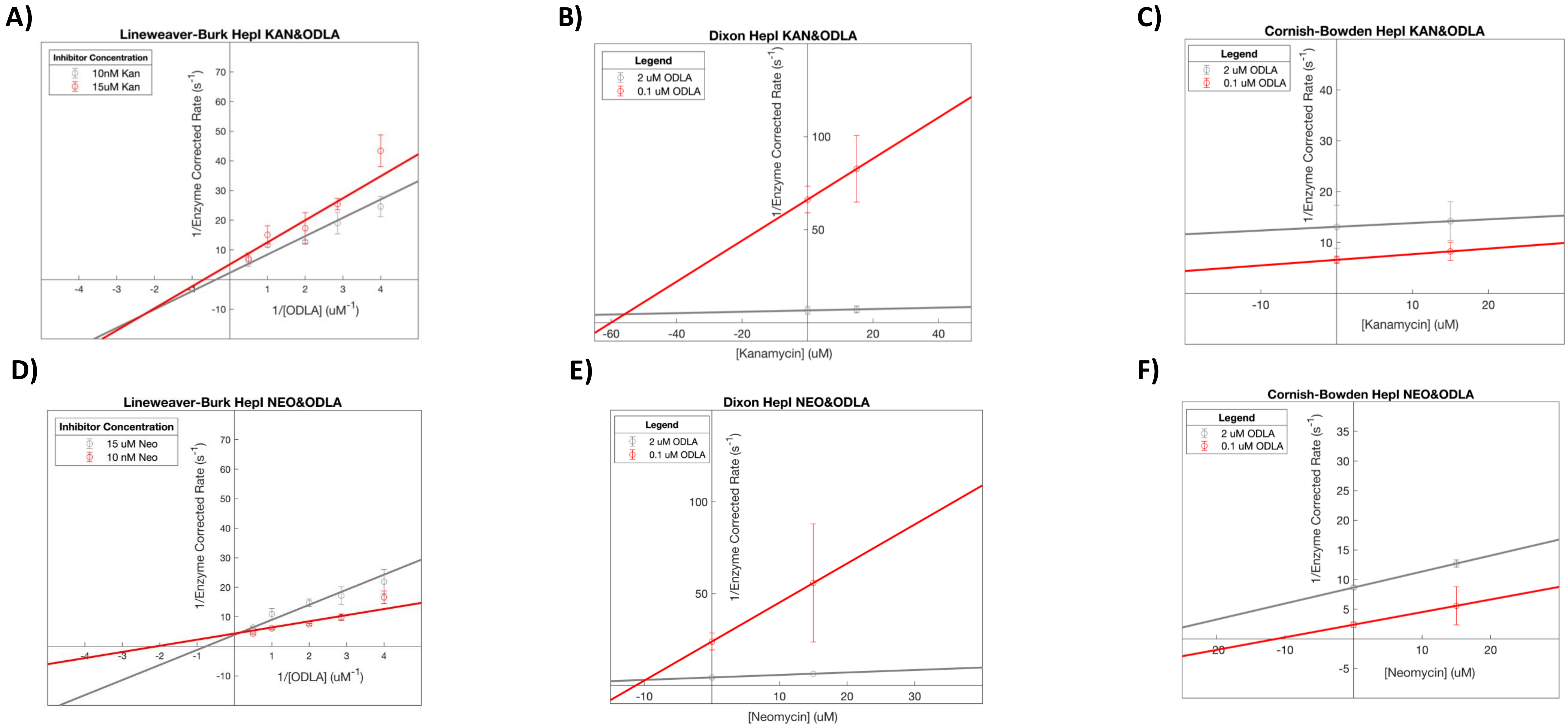

Supplemental Figure 4: Double reciprocal plots of HepI kinetics in the presence of ODLA with varying **A-C)** Kanamycin B or **D-F)** Neomycin.

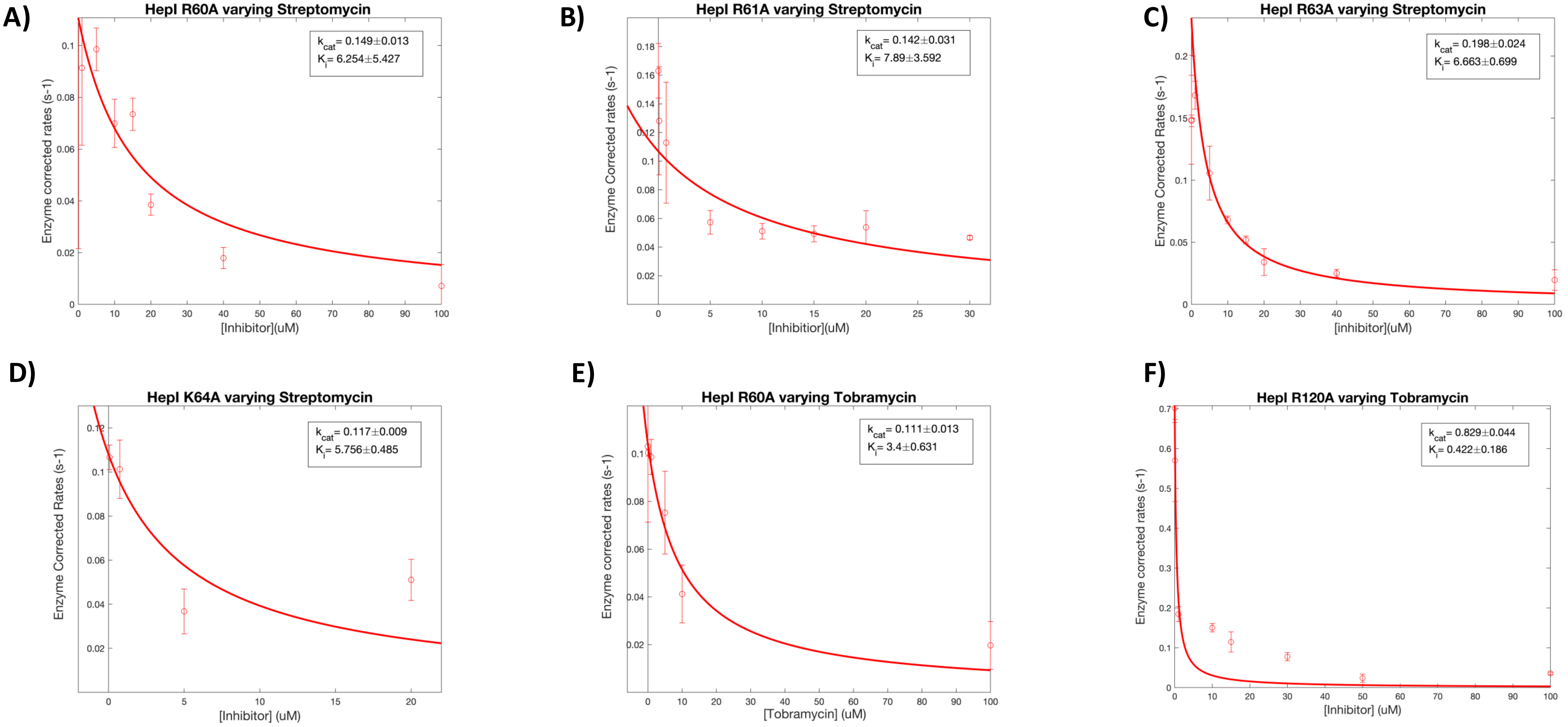

Supplemental Figure 5: Inhibition kinetic curves for HepI mutants **A)** R60A, **B)** R61A, **C)** R63A, **D)** and K64A in the presence of streptomycin. HepI inhibition kinetic curves for **E)** R60A, and **F)** R120A in the presence of tobramycin.

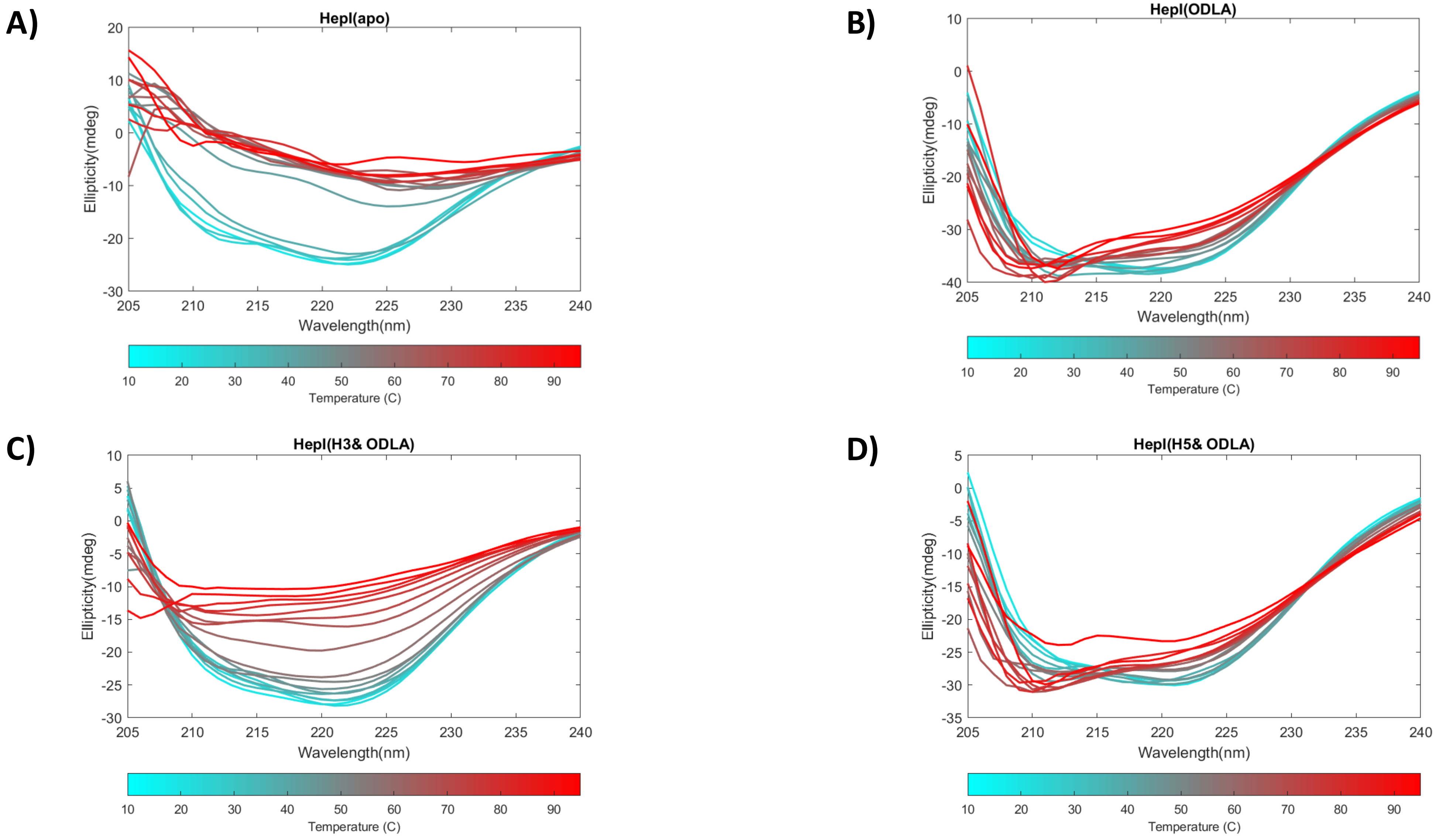

Supplemental Figure 6: CD melts of HepI **A)** apo or in the presence of **B)** ODLA, **C)** ODLA/tobramycin, and **D)** ODLA/streptomycin.

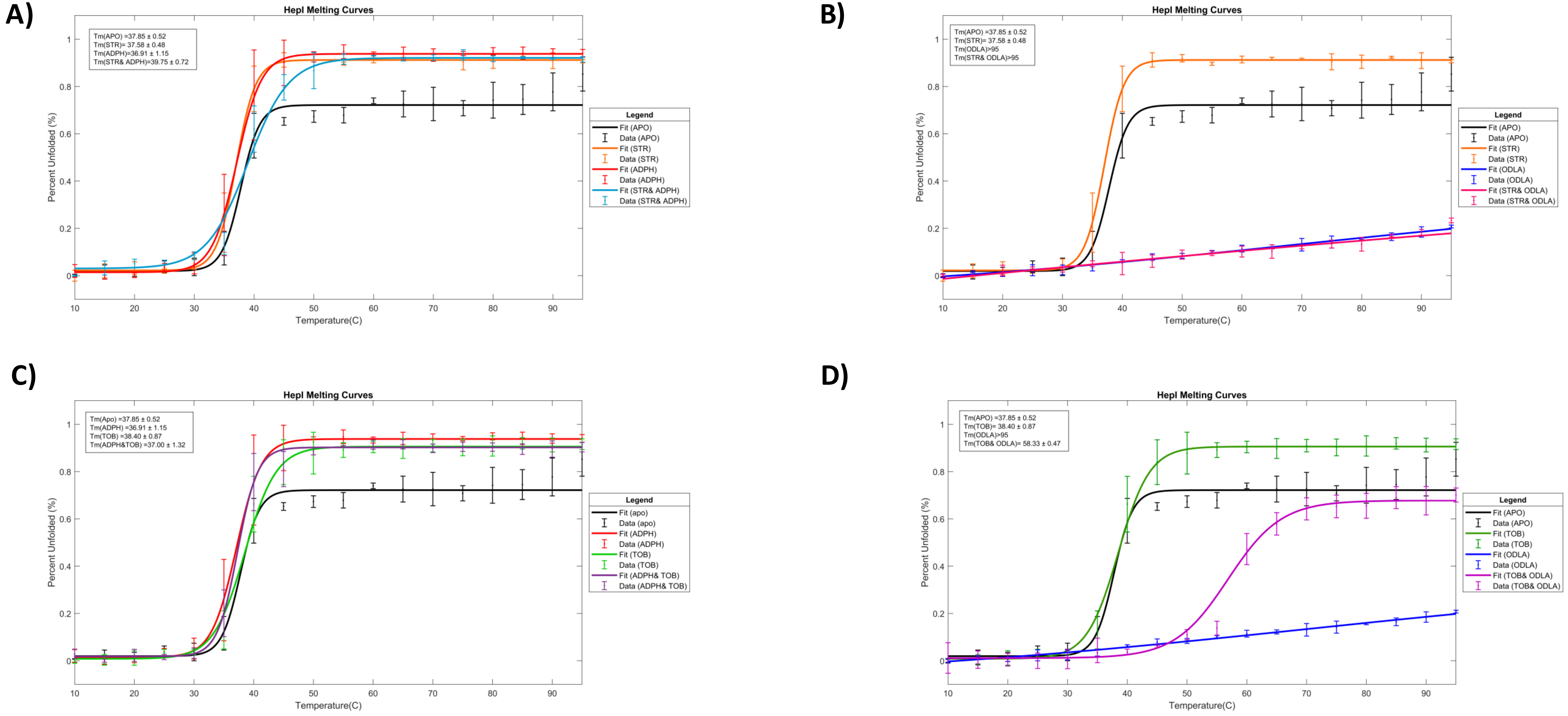

Supplemental Figure 7: Wildtype HepI unfolding monitored via CD at 222 nm across several temperatures for **A)** streptomycin with ADPH, **B)** streptomycin with ODLA, **C)** tobramycin with ADPH and **D)** tobramycin with ODLA.

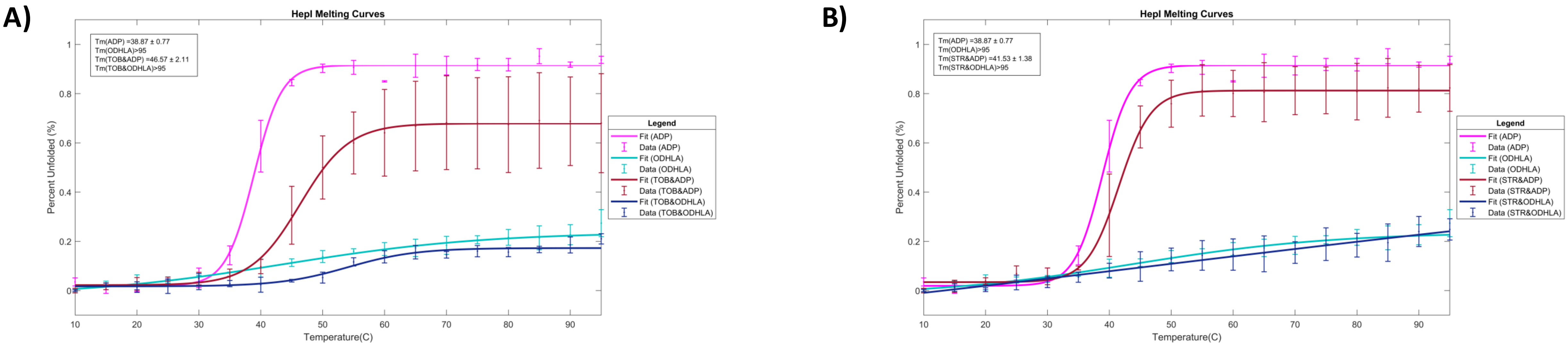

Supplemental Figure 8: HepI unfolding monitored via CD at 222 nm across several temperatures for **A)** tobramycin with products and **B)** streptomycin with products.

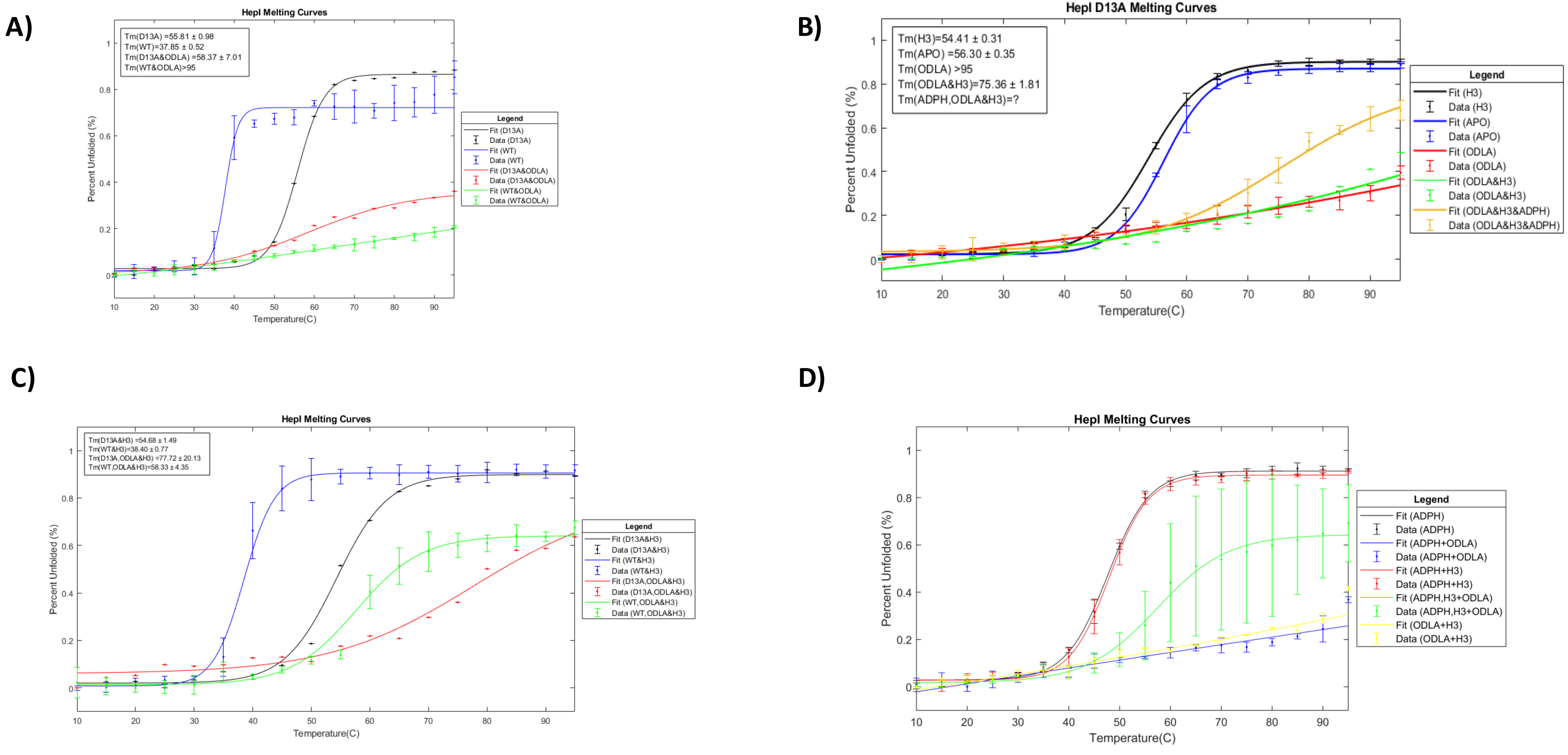

Supplemental Figure 9: HepI unfolding monitored via CD at 222 nm across several temperatures for **A)** wildtype and D13A with substrates, **B)** D13A with substrates and tobramycin, **C)** D13A with substrates and tobramycin and **D)** D13A with substrates and tobramycin.

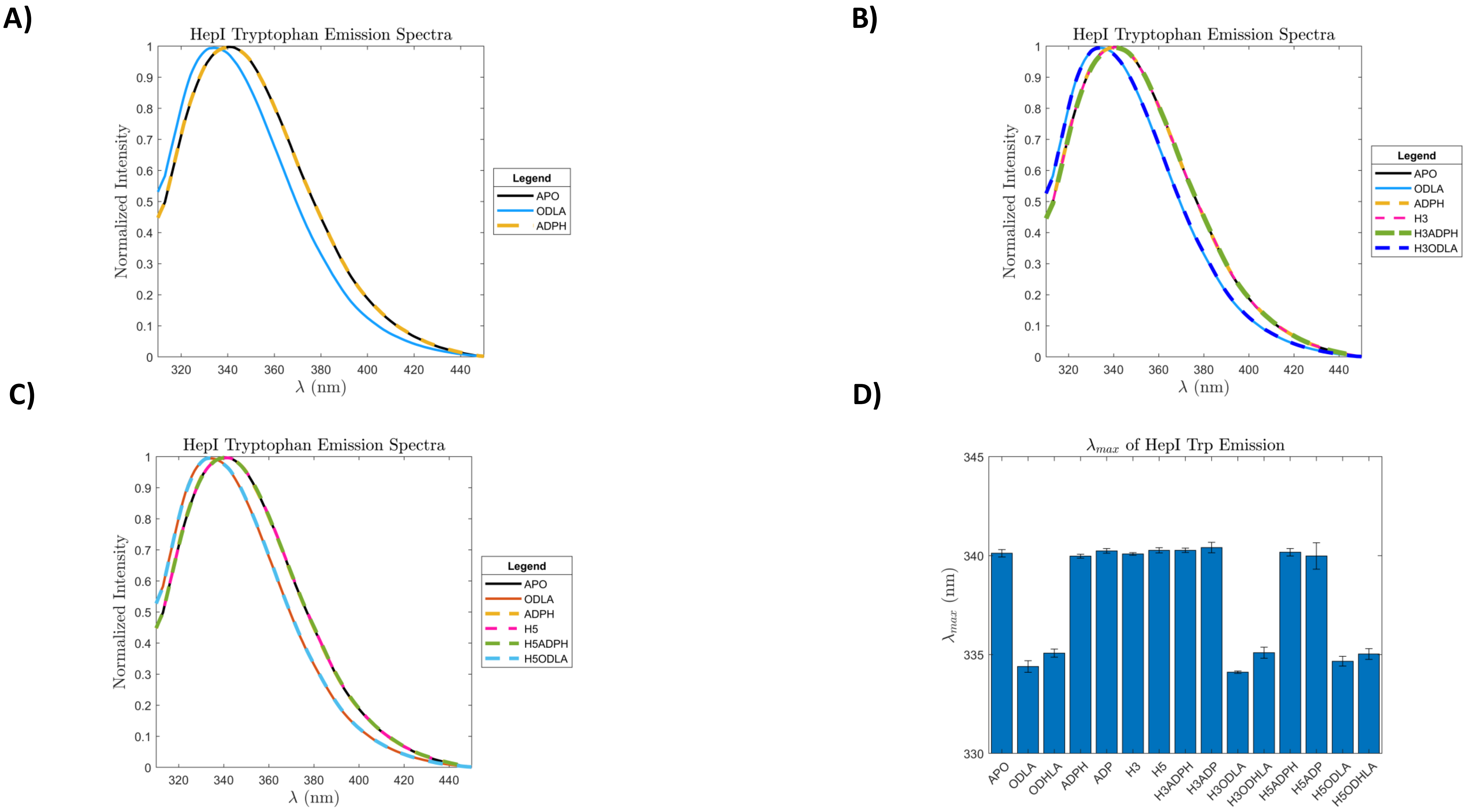

Supplemental Figure 10: HepI tryptophan fluorescence emission spectra for **A)** substrates, **B)** with tobramycin, **C)** with streptomycin, and **B)** λ_{max} in the presence/absence of substrates, products and inhibitors.

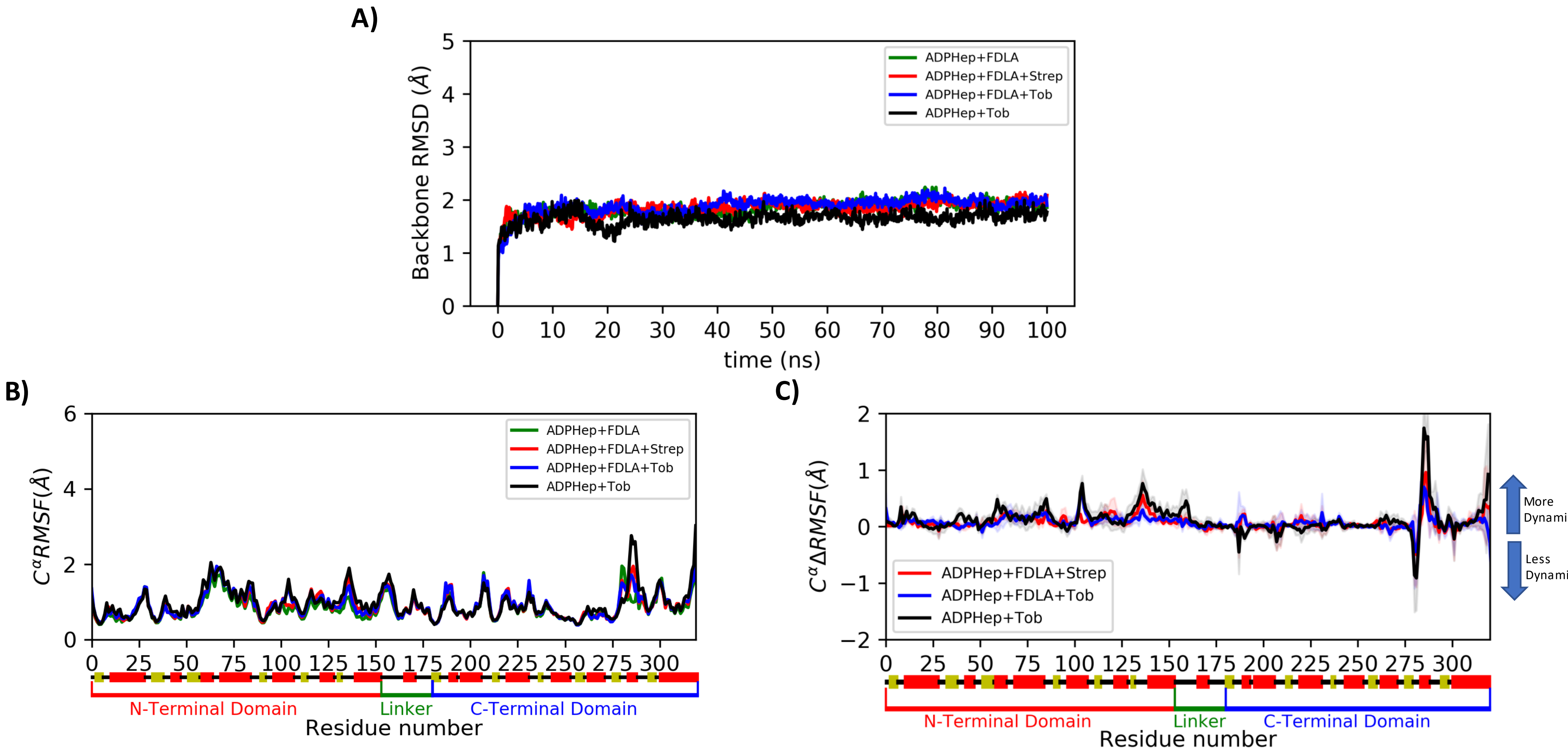

Supplemental Figure 11: (**A**) Backbone RMSD, (**B**) C_{α} RMSF and (**C**) C_{α} ΔRMSF of HepI substrate complex (green), in the presence of Streptomycin (red) or Tobramycin (blue) and HepI binary complex in the presence of Tobramycin(black). Secondary structure of residues are indicated by color (red:α-helix, yellow:β-sheet, black:random coil) below their respective positions.

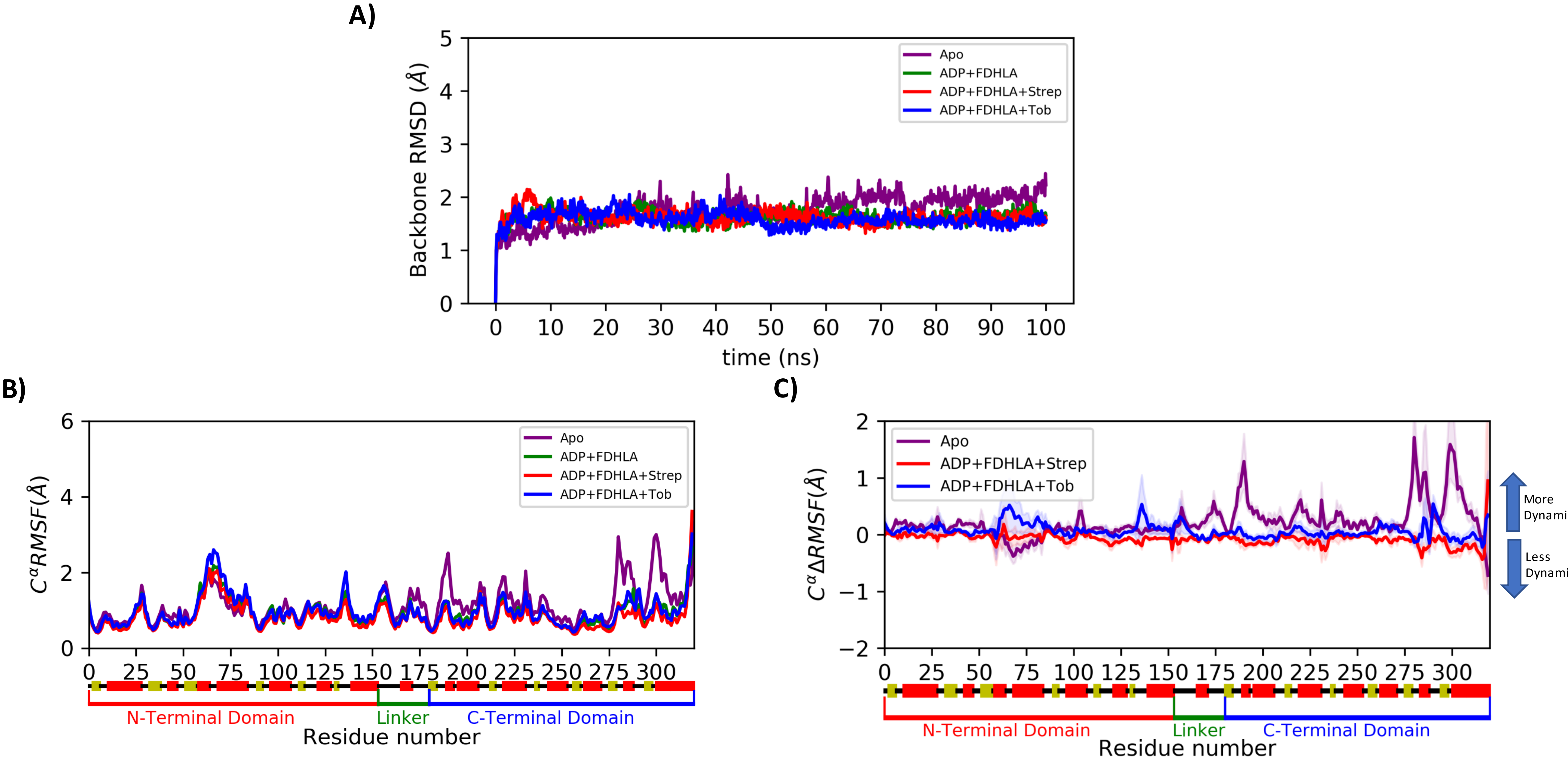

Supplemental Figure 12: (**A**) Backbone RMSD, (**B**) C_{α} RMSF and (**C**) C_{α} ΔRMSF of HepI apo (purple), substrates (green), in the presence of Streptomycin (red) or Tobramycin (blue). Secondary structure of residues are indicated by color (red:α-helix, yellow:β-sheet, black:random coil) below their respective positions.

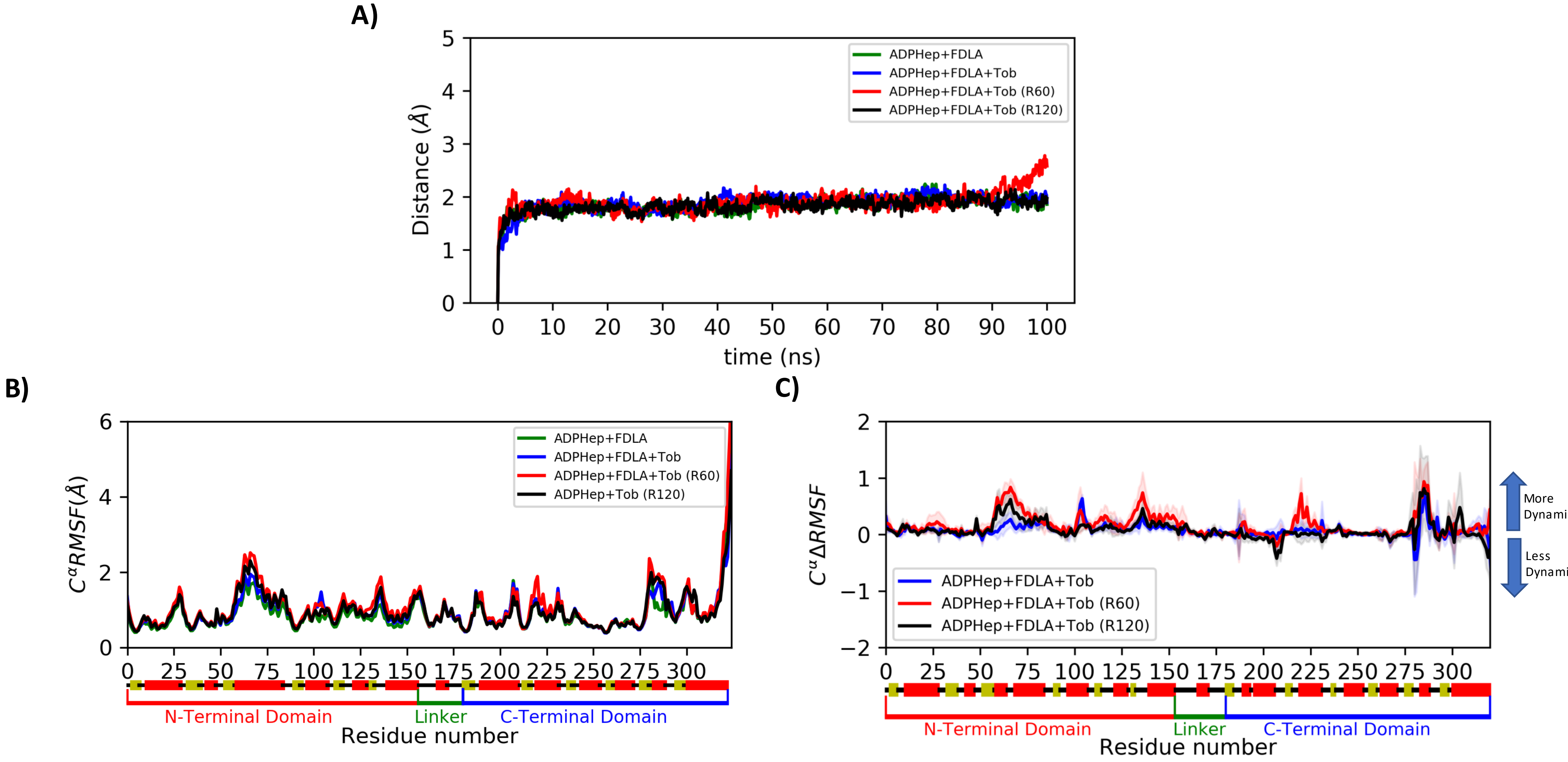

Supplemental Figure 13: (**A**) Backbone RMSD, (**B**) C_α RMSF and (**C**) C_α ΔRMSF of HepI (WT) with substrates (green), in the presence of Tobramycin (blue), HepI (Arg60A) with substrates in the presence of Tobramycin (red), HepI (Arg120A) with substrates in the presence of Tobramycin (black) . Secondary structure of residues are indicated by color (red:α-helix, yellow:β-sheet, black:random coil) below their respective positions.

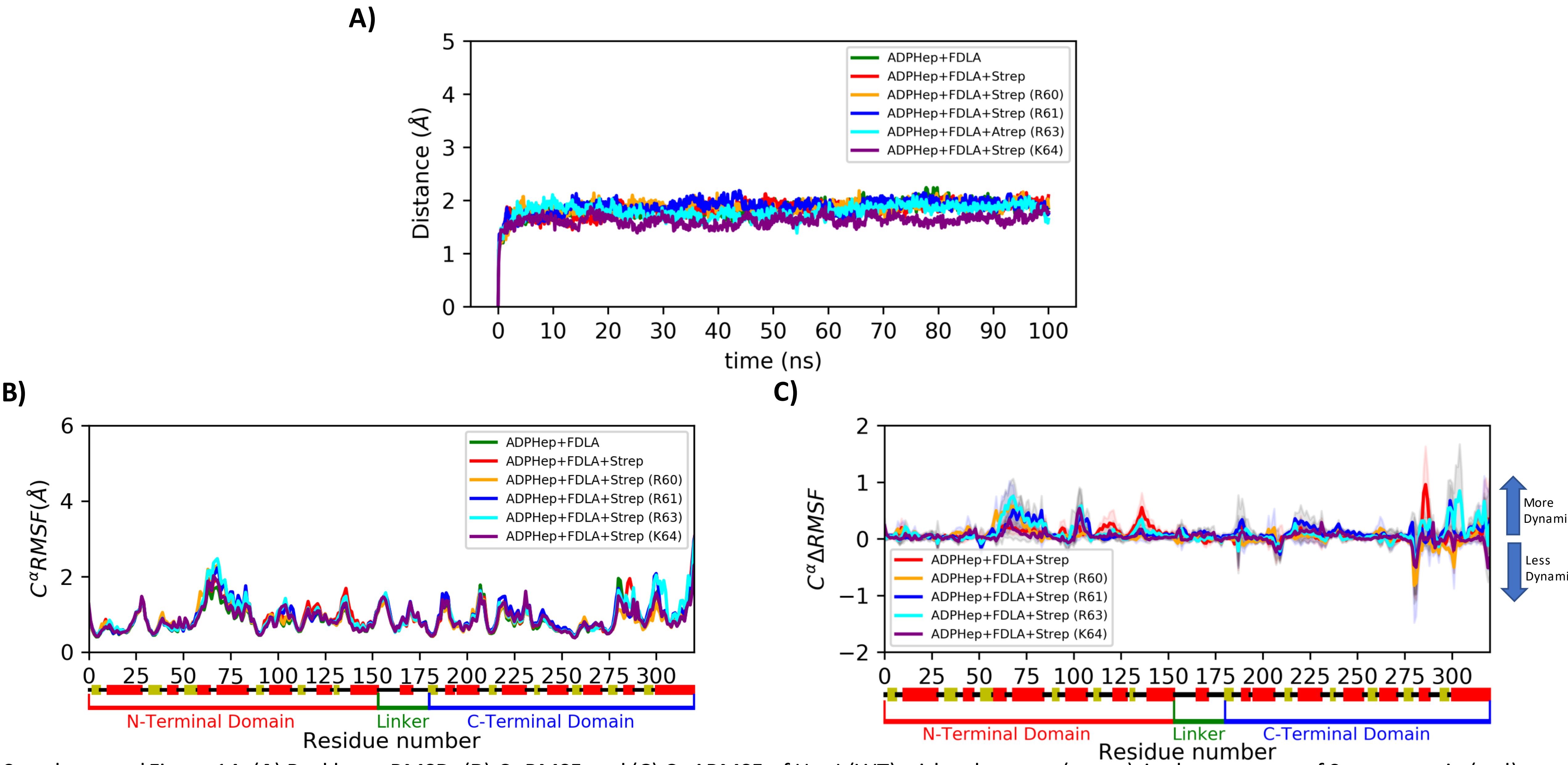

Supplemental Figure 14: (**A**) Backbone RMSD, (**B**) C_{α} RMSF and (**C**) C_{α} ΔRMSF of HepI (WT) with substrates (green), in the presence of Streptomycin (red), HepI (Arg60A) with substrates in the presence of Streptomycin (orange), HepI (Arg61A) with substrates in the presence of Streptomycin (blue), HepI (Arg63A) with substrates in the presence of Streptomycin (cyan), and HepI (Lys64A) with substrates in the presence of Streptomycin (purple) . Secondary structure of residues are indicated by color (red:α-helix, yellow:β-sheet, black:random coil) below their respective positions.

A)

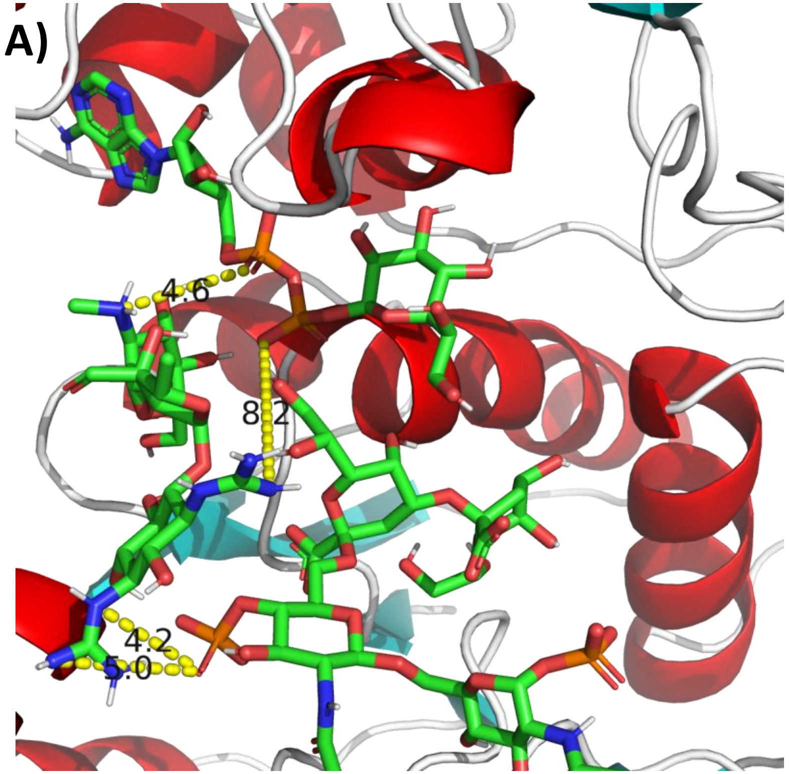

B)

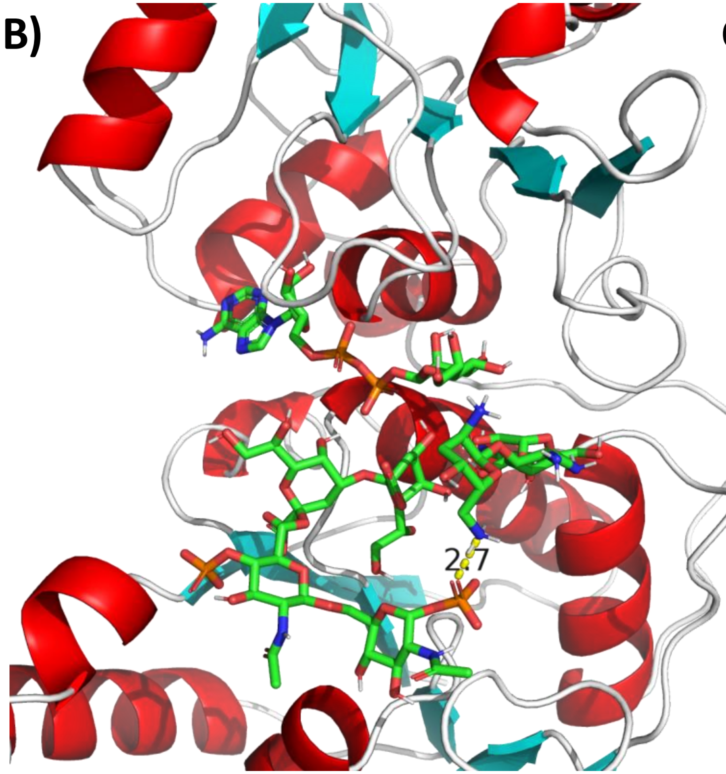

C)

ADPHep	FDLA	Streptomycin	Tobramycin	Average Distance (Å)
Pα (-PO-)		C2’’(-NH2+-)		3.7 ± 1.0
Pβ(-PO-)		C8’(-NH2)		7.6 ± 0.9
	C4 Glcnac(-PO-)	C7’(=NH+-)		6.8 ± 1.1
	C4 Glcnac (-PO-)	C7’(-NH2)		6.8 ± 1.5
	C1 Glcnac (-PO-)		C6’’ (-NH3+)	4.5 ± 0.9

Supplemental Figure 15: (**A**) Model of interactions between ADPHep/FDLA with Streptomycin and (**B**) model of interactions between ADPHep/FDLA with Tobramycin and **C**) average distance of these interactions across three independent trajectories.

A)

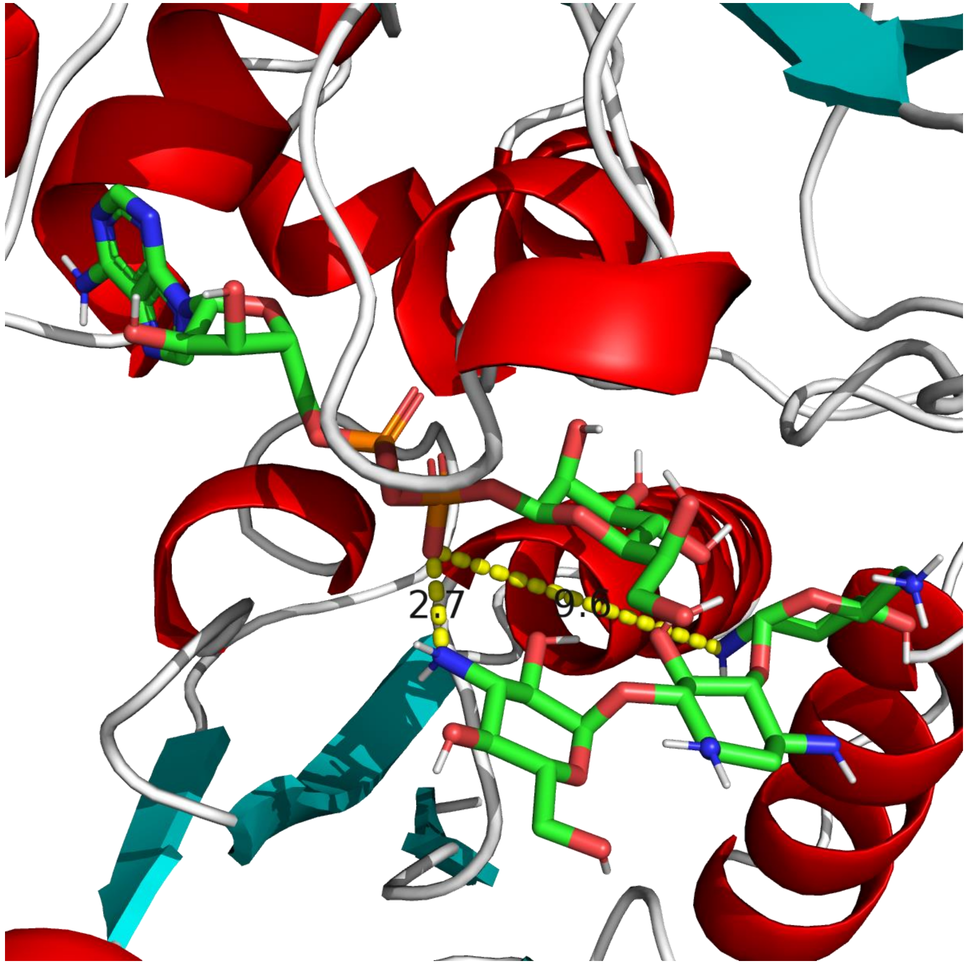

B)

ADPHep	Tobramycin	Average Distance (Å)
O1B (-PO-)	N4 (-NH3+)	3.3 ± 0.3
O1B (-PO-)	N3(-NH3+)	8.8 ± 0.4
O3(-OH)	O4’(-OH)	2.8 ± 0.1

Supplemental Figure 16: (**A**) Representative model and **(B)** table of interactions between ADPHep with Tobramycin across three independent trajectories.

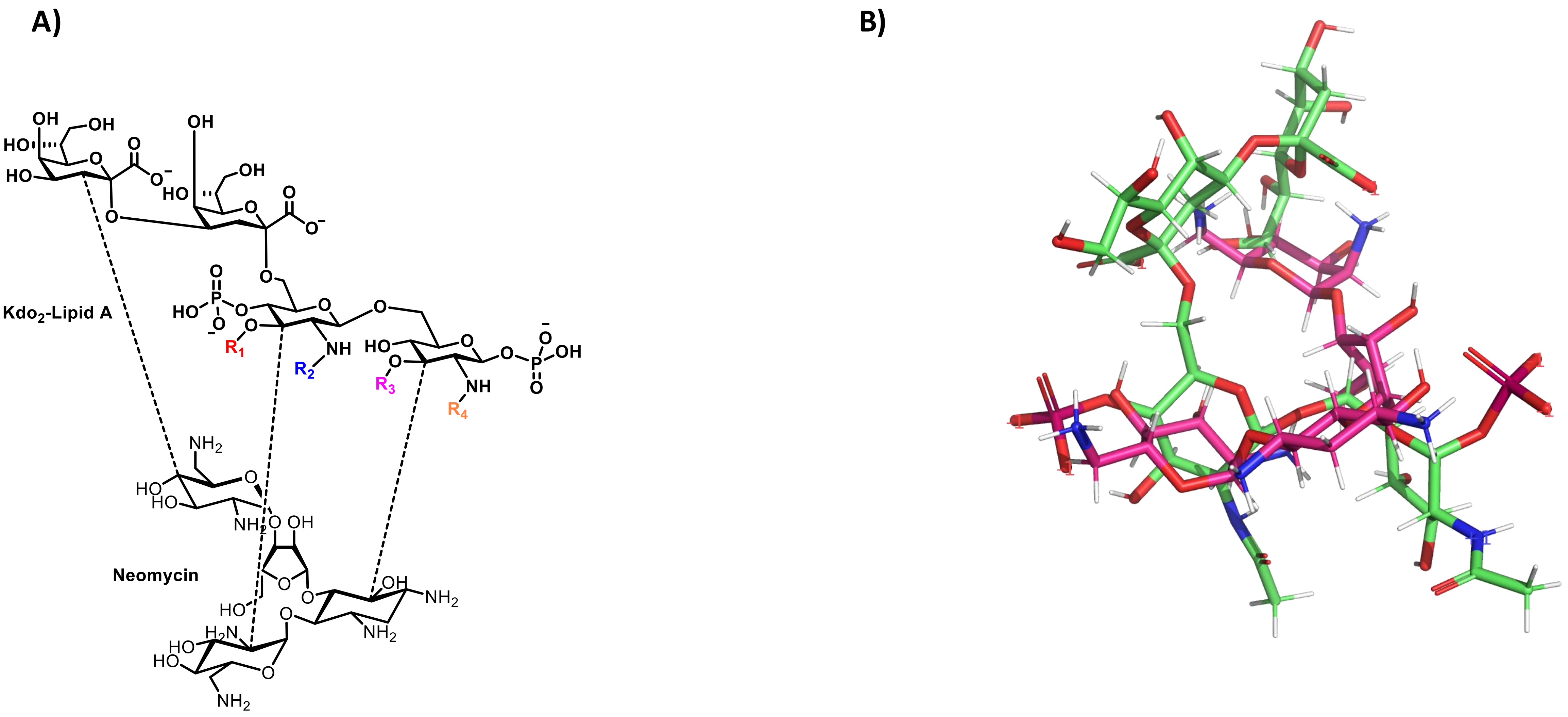

Supplemental Figure 17: (**A**) 3D and **(B)** 2D Structural superposition of Neomycin on fully deacylated donor substrate that neomycin acts as competitive inhibitor towards. Dotted lines show which sugars align with another.

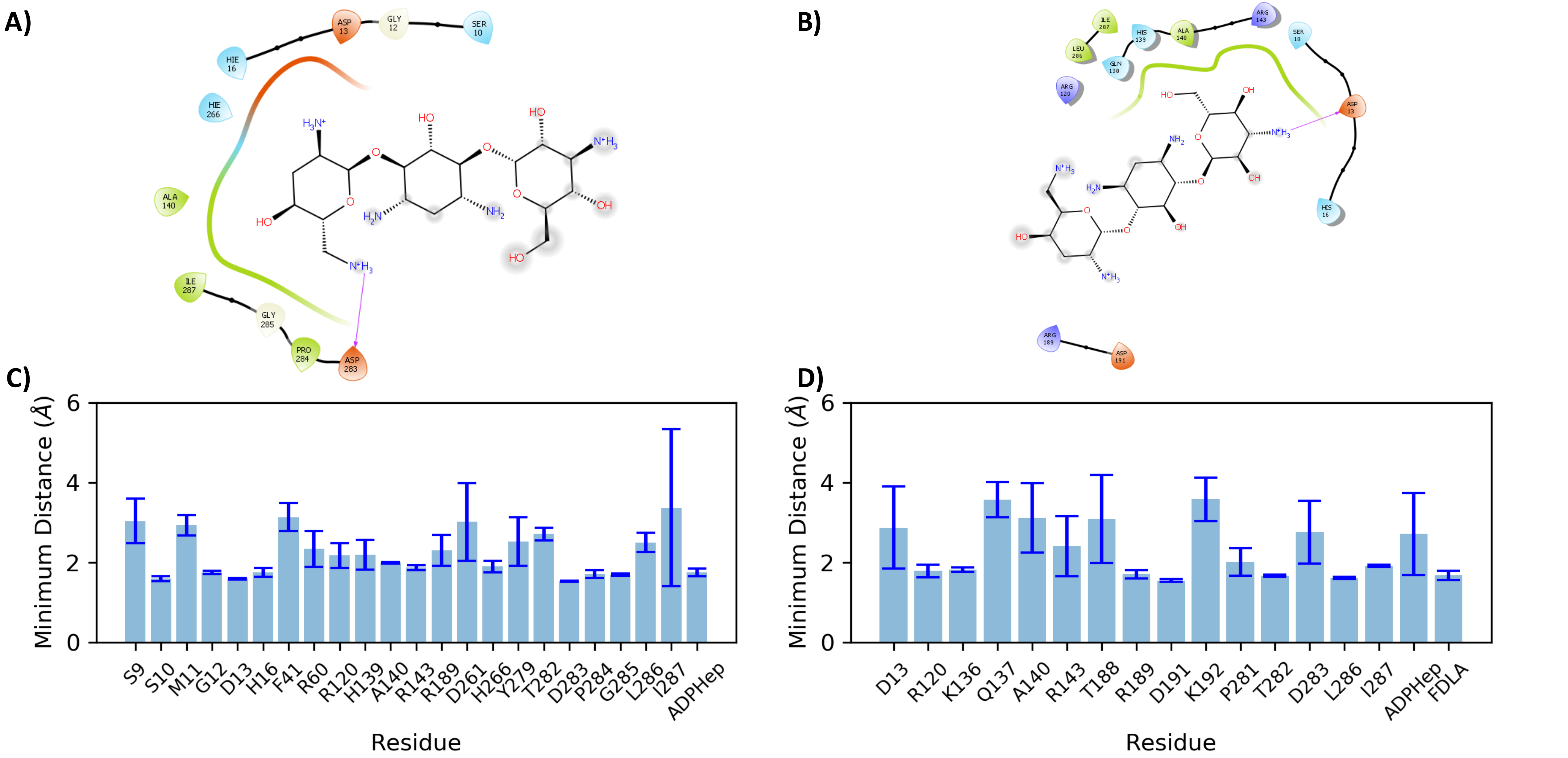

Supplemental Figure 18: (**A**) Tobramycin (binary) Ligand Interaction Diagram (**B**) Tobramycin (ternary) Ligand Interaction Diagram (**C**) Residues with minimum distance <3.5 Å from Tobramycin (binary) and (**D**) Residues with minimum distance <3.5 Å from Tobramycin (ternary)

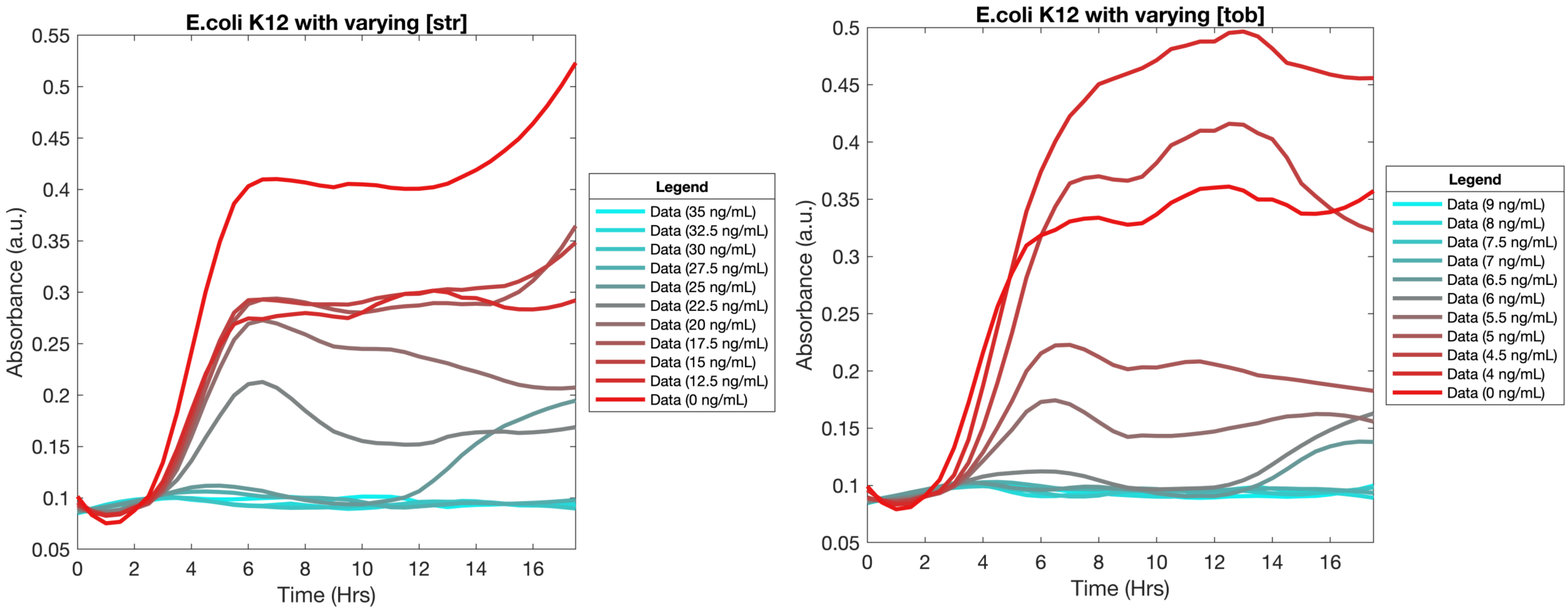

Supplemental Figure 19: *E. coli* K12 growth patterns with varying concentrations of (A) streptomycin and (B) tobramycin over 18 hrs

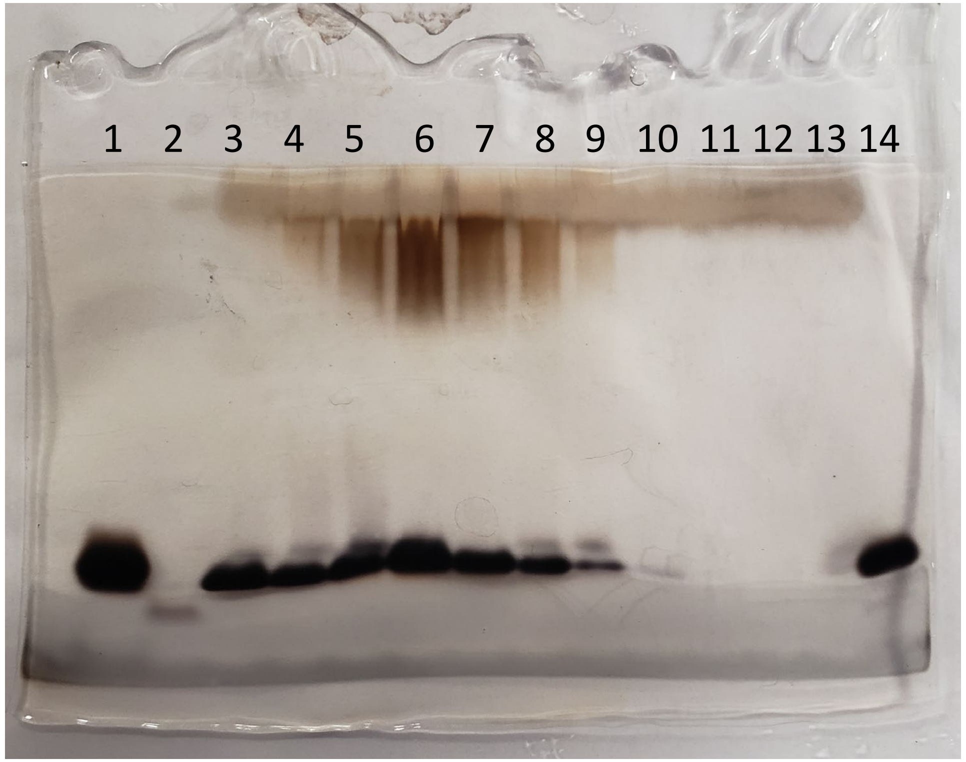

Lanes
1: Heptosylated Lipid A
2: Lipid A
3: 0 ng/mL TOB
4: 4 ng/mL TOB
5: 4.5 ng/mL TOB
6: 5 ng/mL TOB
7: 5.5 ng/mL TOB
8: 6 ng/mL TOB
9: 6.5 ng/mL TOB
10: 7 ng/mL TOB
11: 7.5 ng/mL TOB
12: 8 ng/mL TOB
13: 9 ng/mL TOB
14: : Heptosylated Lipid A

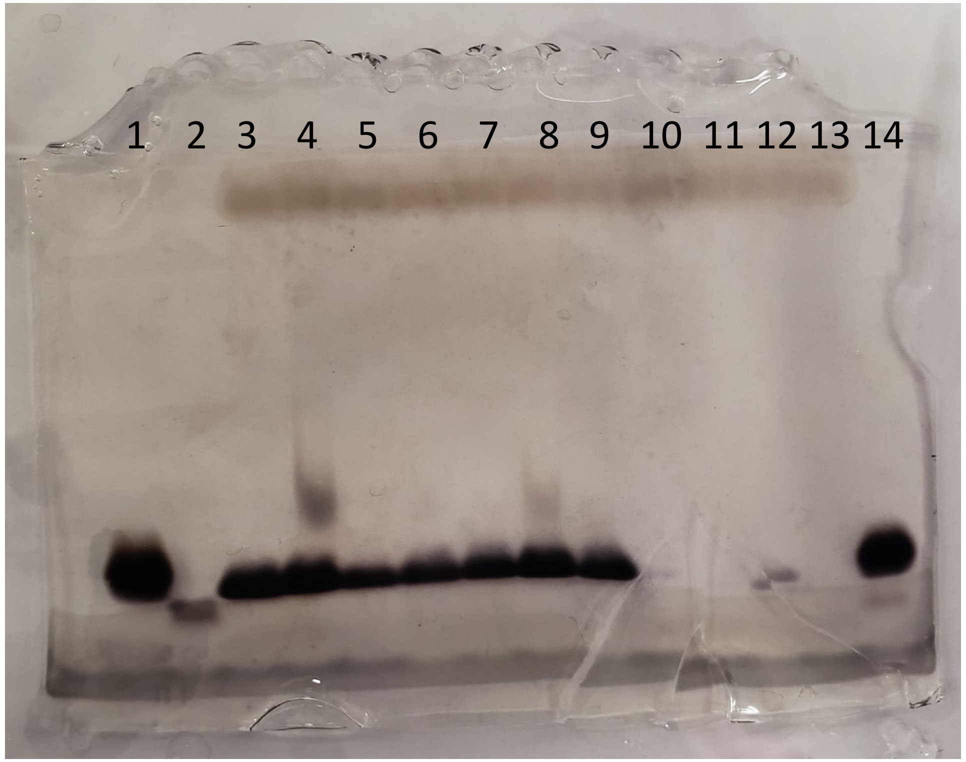

Lanes
1: Heptosylated Lipid A
2: Lipid A
3: 0 ng/mL STR
4: 12 ng/mL STR
5: 15 ng/mL STR
6: 17.5 ng/mL STR
7: 20 ng/mL STR
8: 22.5 ng/mL STR
9: 25 ng/mL STR
10: 27.5 ng/mL STR
11: 30 ng/mL STR
12: 32.5 ng/mL STR
13: 35 ng/mL STR
14: : Heptosylated Lipid A

Supplemental Figure 20: 6%/16% polyacrylamide gels silver stained, with total lipid extracts from *E. coli* K12 grown at various concentrations of (A) tobramycin and (B) streptomycin.

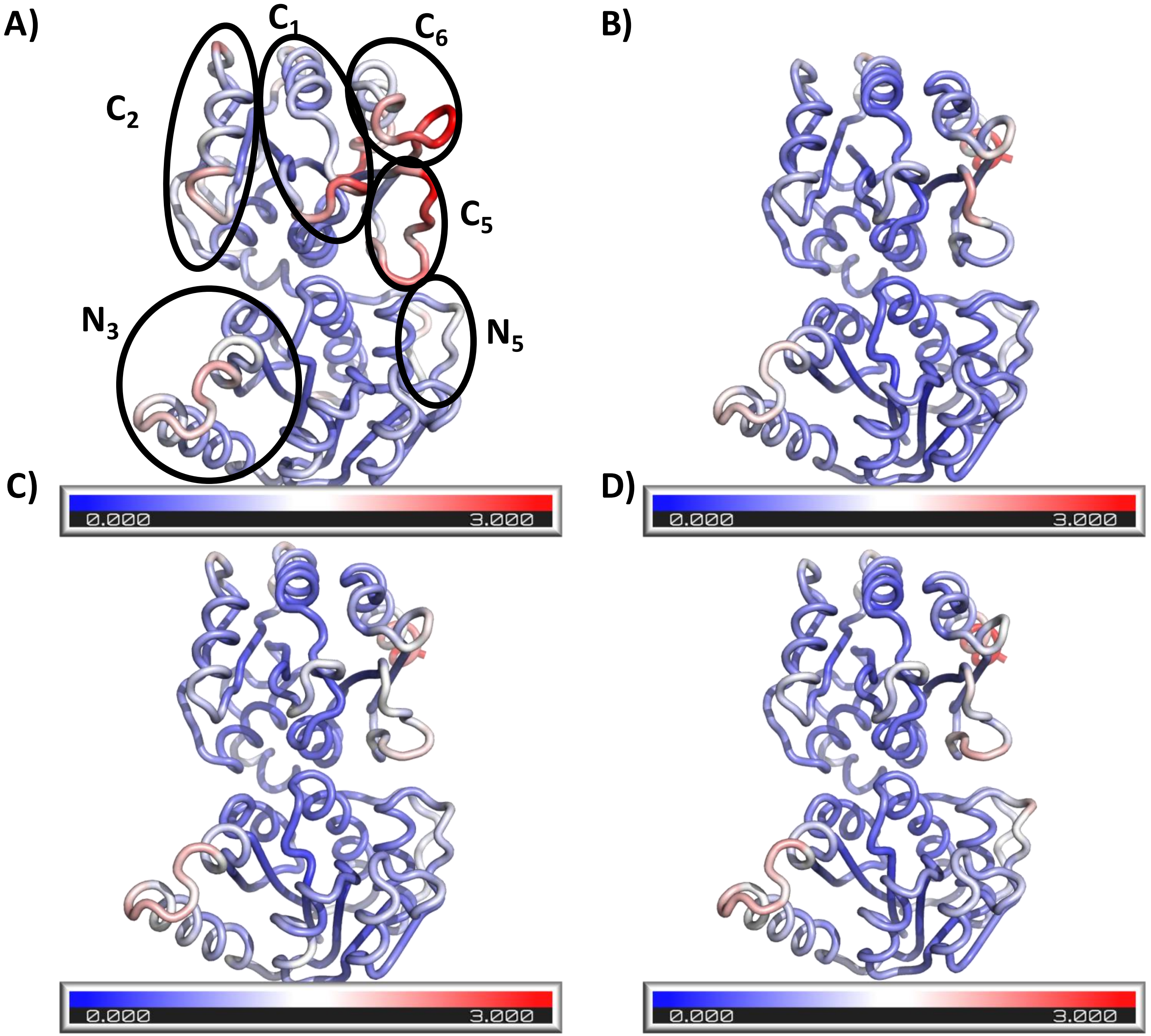

Supplemental Figure 21: RMSF for HepI (**A**) apo, (**B**) substrates ternary complex, (**C**) substrate ternary complex with tobramycin and (**D**) substrate ternary complex with Streptomycin mapped onto the structures and colored from least dynamic (blue) to most dynamic (red) with a cutoff of 3.0 Å

A)

<table>
<tr><th>Simulation</th><th>PDB Code</th><th>Mutation</th><th>ADP-Hep</th><th>FDLA</th><th>ADP</th><th>FDHLA</th><th>Tob</th><th>Strep</th></tr>
<tr><td>HepI</td><td>2GT1</td><td>WT</td><td colspan="6">N/A</td></tr>
<tr><td>ADP-Hep•FDLA•HepI</td><td>6DFE</td><td>WT</td><td>Added</td><td>Present</td><td colspan="4">N/A</td></tr>
<tr><td>ADP•FDHLA•HepI</td><td>6DFE</td><td>WT</td><td colspan="2">N/A</td><td>Added</td><td>Modified</td><td colspan="2">N/A</td></tr>
<tr><td>Tob•ADP-Hep•FDLA•HepI</td><td>6DFE</td><td>WT</td><td>Added</td><td>Present</td><td colspan="2">N/A</td><td>Docked</td><td>N/A</td></tr>
<tr><td>Strep•ADP-Hep•FDLA•HepI</td><td>6DFE</td><td>WT</td><td>Added</td><td>Present</td><td colspan="2">N/A</td><td>N/A</td><td>Docked</td></tr>
<tr><td>Tob•ADP-Hep•HepI</td><td>2H1H</td><td>WT</td><td>Modified</td><td>N/A</td><td colspan="2">N/A</td><td>Docked</td><td>N/A</td></tr>
<tr><td>Tob•ADP•FDHLA•HepI</td><td>6DFE</td><td>WT</td><td colspan="2">N/A</td><td>Added</td><td>Modified</td><td>Docked</td><td>N/A</td></tr>
<tr><td>Strep•ADP•FDHLA•HepI</td><td>6DFE</td><td>WT</td><td colspan="2">N/A</td><td>Added</td><td>Modified</td><td>N/A</td><td>Docked</td></tr>
<tr><td>Tob•ADP-Hep•FDLA•HepI</td><td>6DFE</td><td>Arg60Ala</td><td>Added</td><td>Present</td><td colspan="2">N/A</td><td>Docked</td><td>N/A</td></tr>
<tr><td>Tob•ADP-Hep•FDLA•HepI</td><td>6DFE</td><td>Arg120Ala</td><td>Added</td><td>Present</td><td colspan="2">N/A</td><td>Docked</td><td>N/A</td></tr>
<tr><td>Strep•ADP-Hep•FDLA•HepI</td><td>6DFE</td><td>Arg60Ala</td><td>Added</td><td>Present</td><td colspan="2">N/A</td><td>N/A</td><td>Docked</td></tr>
<tr><td>Strep•ADP-Hep•FDLA•HepI</td><td>6DFE</td><td>Arg61Ala</td><td>Added</td><td>Present</td><td colspan="2">N/A</td><td>N/A</td><td>Docked</td></tr>
<tr><td>Strep•ADP-Hep•FDLA•HepI</td><td>6DFE</td><td>Arg63Ala</td><td>Added</td><td>Present</td><td colspan="2">N/A</td><td>N/A</td><td>Docked</td></tr>
<tr><td>Strep•ADP-Hep•FDLA•HepI</td><td>6DFE</td><td>Lys64Ala</td><td>Added</td><td>Present</td><td colspan="2">N/A</td><td>N/A</td><td>Docked</td></tr>
</table>

Supplemental Table 1: The simulations performed in this study including PDB codes of crystal structures that were used and whether ligands were present in the original structure, added into position based on information from another crystal structure, modified atoms to mimic the native ligands, docked or if any mutations were introduced into the structure.

Simulation	Mutation	RMSD (Å)	R_{gyr} (Å)	Interdomain Distance (Å)	RMSF (Residues >1.5 Å)
HepI	WT	1.76 ± 0.45	21.32 ± 0.24	30.26 ± 0.67	29, 60, 63, 64, 65, 66, 67, 68, 69, 70, 157, 158, 171, 175, 188, 189, 190, 191, 192, 208, 210, 218, 219, 220, 221, 232, 280, 281, 282, 283, 284, 285, 286, 287, 288, 289, 298, 299, 300, 301, 302, 303, 304, 305, 306, 317, 318, 319, 320, 321, 322, 323
ADP-Hep•FDLA•HepI	WT	1.84 ± 0.23	21.13 ± 0.16	29.74 ± 0.43	64, 65, 67, 68, 69, 70, 208, 210, 281, 282, 283, 301, 302, 319, 320, 321, 322, 323, 324, 325
ADP•FDHLA•HepI	WT	1.62 ± 0.26	21.09 ± 0.16	28.24 ± 0.38	60, 61, 63, 64, 65, 66, 67, 68, 69, 70, 71, 72, 73, 84, 137, 289, 317, 318, 319, 320
Tob•ADP-Hep•FDLA•HepI	WT	1.88 ± 0.30	21.23 ± 0.12	30.05 ± 0.37	63, 64, 65, 66, 67, 68, 69, 71, 187, 207, 209, 231, 280, 284, 285, 286, 300, 301, 317, 318, 319, 320, 321, 322, 323, 324
Strep•ADP-Hep•FDLA•HepI	WT	1.84 ± 0.25	21.28 ± 0.16	30.28 ± 0.47	62, 63, 64, 65, 66, 67, 68, 69, 72, 135, 136, 207, 280, 285, 286, 287, 288, 300, 317, 318, 319, 320, 321, 322, 323, 324
Tob•ADP-Hep•HepI	WT	1.65 ± 0.30	21.26 ± 0.19	29.53 ± 0.56	59, 60, 62, 63, 64, 65, 66, 67, 68, 69, 72, 83, 84, 85, 104, 134, 135, 136, 137, 156, 157, 158, 207, 284, 285, 286, 287, 288, 300, 317, 318, 319
Tob•ADP•FDHLA•HepI	WT	1.59 ± 0.33	21.32 ± 0.9	28.76 ± 0.51	61, 62, 63, 64, 65, 66, 67, 68, 69, 70, 71, 72, 73, 74, 76, 77, 84, 136, 137, 156, 157, 158, 286, 291, 292, 318, 319, 320
Strep•ADP•FDHLA•HepI	WT	1.62 ± 0.30	21.16 ± 0.14	28.29 ± 0.34	63 , 64, 65, 66, 67, 68, 69, 70, 71, 72, 73, 318, 319 ,320
Tob•ADP-Hep•FDLA•HepI	Arg60Ala	1.90 ± 0.28	21.38 ± 0.18	30.45 ± 0.52	28, 59, 60, 61, 62, 63, 64, 65, 66, 67, 68, 69, 70, 71, 72, 73, 75, 76, 134, 135, 136, 137, 154, 157, 207, 219, 220, 280, 281, 282, 283, 284, 285, 286, 287, 288, 300, 301, 318, 319, 320, 321, 322, 323, 324
Tob•ADP-Hep•FDLA•HepI	Arg120Ala	1.83 ± 0.33	21.29 ± 0.19	30.19 ± 0.53	59, 60, 62, 63, 64, 65, 66, 67, 68, 69, 70, 71, 72, 83, 136, 280, 281, 282, 283, 284, 285, 286, 287, 288, 299, 300, 304, 319, 320, 321, 322, 323, 324
Strep•ADP-Hep•FDLA•HepI	Arg60Ala	1.93 ± 0.23	21.17 ± 0.16	29.93 ± 0.50	59, 60, 61, 62, 63, 64, 65, 66, 67, 68, 69, 70, 71, 72, 207, 317, 318, 319, 320, 321, 322, 323, 324
Strep•ADP-Hep•FDLA•HepI	Arg61Ala	1.88 ± 0.34	21.21 ± 0.14	30.10 ± 0.38	63, 64, 65, 66, 67, 68, 69, 70, 71, 72, 73, 75, 76, 79, 83, 157, 187, 189, 207, 280, 281, 288, 299, 300, 301, 317, 318, 319, 320, 321, 322, 323, 324
Strep•ADP-Hep•FDLA•HepI	Arg63Ala	1.76 ± 0.22	21.26 ± 0.16	30.15 ± 0.37	62, 63, 64, 65, 66, 67, 68, 69, 70, 71, 72, 73, 75, 83, 157, 207, 280, 281, 284, 288, 298, 299, 300, 301, 302, 303, 304, 305, 317, 318, 319, 320, 321, 322, 323, 324
Strep•ADP-Hep•FDLA•HepI	Lys64Ala	1.61 ± 0.19	21.12 ± 0.12	29.80 ± 0.36	63, 64, 65, 66, 67, 68, 69, 207, 231, 300, 319, 320, 321, 322, 323, 324

Supplemental Table 2: Average values of RMSD, and radius of gyration, interdomain center of mass distance. RMSF values are reported for residues that have a greater than 1.5 Å fluctuation.

Inhibitor	Mutation	ΔG_{Exp} (kcal/mol)	ΔG_{Calc} (kcal/mol)	$\Delta\Delta G_{Exp}$ (kcal/mol)	$\Delta\Delta G_{Calc}$ (kcal/mol)	Pearson Correlation Coefficient (R)
Tobramycin	WT	-8.1 ± 0.1	-10.5 ± 5.7	---	---	0.87
	Arg60Ala	-7.5 ± 0.1	-8.1 ± 2.3	0.7 ± 0.1	2.4 ± 6.2	
	Arg120Ala	-8.7 ± 0.2	-13.8 ± 3.5	-0.6 ± 0.2	-3.3 ± 6.7	
Streptomycin	WT	-8.5 ± 0.1	-13.1 ± 5.3	---	---	
	Arg60Ala	-7.1 ± 0.4	-9.9 ± 3.1	1.4 ± 0.4	3.2 ± 6.1	
	Arg61Ala	-7.0 ± 0.2	-10.9 ± 2.1	1.5 ± 0.2	2.2 ± 5.7	
	Arg63Ala	-7.1 ± 0.1	-8.5 ± 1.5	1.4 ± 0.1	4.6 ± 5.5	
	Lys64Ala	-7.1 ± 0.1	-6.8 ± 7.6	1.3 ± 0.1	6.3 ± 9.2	

Supplemental Table 3: (**A**)Table of binding energies of Streptomycin/Tobramycin to HepI both wildtype and Arg/Lys mutants with correlation values.

[Tobramycin] ng/mL	Relative pixel density from cps band	[Streptomycin] ng/mL	Relative pixel density from cps band
0	1	0	1
4	1.752	12.5	1.019
4.5	4.293	15	1.115
5	7.121	17.5	1.105
5.5	5.786	20	1.323
6	3.692	22.5	1.458
6.5	2.319	25	1.335
7	1.163	27.5	1.687
7.5	1.421	30	1.477
8	1.641	32.5	1.070
9	1.606	35	0.654

Supplemental Table 4: Concentrations of inhibitors tobramycin and streptomycin with the associated pixel density from the capsular polysaccharide band extracted from total lipid extract and resolved using gel electrophoresis.
